# Supplementary material for: The Complete Mitochondrial Genome of Four Hylicinae (Hemiptera: Cicadellidae): Structural Features and Phylogenetic Implications
Source: Insects. 2020 Dec 7;11(12):869. doi: 10.3390/insects11120869 (PMC7762291; doi:10.3390/insects11120869)
Supplement: Supplementary file 1 [file insects-11-00869-s001.pdf]

Table S1. Mtgenome organization of *N. tuberculatus.*

Name	Location		Size(bp)	Intergenic	Codon		Strand
	From	To		nucleotides	Start	Stop	
trnI	1	67	67				J
trnQ	65	135	71	-3			N
trnM	136	206	71	-			J
nad2	207	1181	975	-	ATT	TAG	J
trnW	1180	1247	68	-2			J
trnC	1240	1302	63	-8			N
trnY	1303	1370	68	-			N
cox1	1369	2902	1534	-2	ATG	T	J
trnL2(UUR)	2903	2966	64	-			J
cox2	2967	3645	679	-	ATA	T	J
trnK	3647	3715	69	1			J
trnD	3715	3776	62	-1			J
atp8	3777	3929	153	-	ATA	TAA	J
atp6	3917	4573	657	-13	ATG	TAA	J
cox3	4576	5355	780	2	ATG	TAA	J
trnG	5355	5415	61	-1			J
nad3	5416	5769	354	-	ATT	TAA	J
trnA	5770	5830	61	-			J
trnR	5829	5892	64	-2			J
trnN	5895	5960	66	2			J
trnS1(ACN)	5960	6021	62	-1			J
trnE	6022	6082	61	-			J
trnF	6085	6150	66	2			N
nad5	6151	7813	1663	-	TTG	T	N
trnH	7814	7874	61	-			N
nad4	7878	9225	1348	3	ATT	T	N
nad4L	9206	9472	267	-20	ATT	TAG	N
trnT	9475	9537	63	2			J
trnP	9538	9605	68	-			N
nad6	9608	10091	484	2	ATT	T	J
cytb	10092	11228	1137	-	ATG	TAG	J
trnS2(UCN)	11227	11289	63	-2			J
nad1	11280	12203	924	-10	ATT	TAA	N
trnL1(CUN)	12204	12270	67	-			N
rrnL	12271	13465	1195	-			N
trnV	13466	13528	63	-			N
rrnS	13529	14258	730	-			N
CR	14259	15737	1479	-			J

Table S2. Mtgenome organization of *H. paradoxa.*

Name	Location		Size(bp)	Intergenic	Codon		Strand
	From	To		nucleotides	Start	Stop	
trnI	1	64	64				J
trnQ	62	131	70	-3			N
trnM	131	200	70	-1			J

nad2	201	1172	972	-	ATC	TAG	J
trnW	1171	1235	65	-2			J
trnC	1228	1291	64	-8			N
trnY	1292	1357	66	-			N
cox1	1356	2892	1537	-2	ATG	T	J
trnL2(UUR)	2893	2957	65	-			J
cox2	2958	3636	679	-	ATT	T	J
trnK	3637	3704	68	-			J
trnD	3704	3764	61	-1			J
atp8	3765	3917	153	-	ATA	TAA	J
atp6	3905	4561	657	-13	ATG	TAA	J
cox3	4569	5346	778	7	ATG	T	J
trnG	5347	5408	62	-			J
nad3	5409	5762	354	-	ATA	TAA	J
trnA	5763	5823	61	-			J
trnR	5826	5889	64	2			J
trnN	5891	5957	67	1			J
trnS1(ACN)	5957	6017	61	-1			J
trnE	6017	6078	62	-1			J
trnF	6078	6143	66	-1			N
nad5	6144	7803	1660	-	TTG	T	N
trnH	7804	7866	63	-			N
nad4	7870	9190	1321	3	ATT	T	N
nad4L	9171	9434	264	-20	ATT	TAG	N
trnT	9437	9502	66	2			J
trnP	9503	9567	65	-			N
nad6	9570	10053	484	2	ATT	T	J
cytb	10054	11190	1137	-	ATG	TAG	J
trnS2(UCN)	11194	11255	62	3			J
nad1	11246	12187	942	-10	ATA	TAA	N
trnL1(CUN)	12188	12250	63	-			N
rrnL	12251	13438	1188	-			N
trnV	13439	13499	61	-			N
rrnS	13500	14231	732	-			N
CR	14232	14762	531	-			J

Table S3. Mtgenome organization of *B. fujiana*.

Name	Location		Size(bp)	Intergenic	Codon		Strand
	From	To		nucleotides	Start	Stop	
trnI	1	63	63				J
trnQ	61	128	68	-3			N
trnM	135	202	68	6			J
nad2	203	1177	975	-	ATA	TAA	J
trnW	1176	1236	61	-2			J
trnC	1229	1289	61	-8			N
trnY	1290	1356	67				N
cox1	1355	2888	1534	-2	ATG	T	J
trnL2(UUR)	2889	2958	70	-			J
cox2	2959	3637	679	-	ATA	T	J
trnK	3638	3706	69	-			J

trnD	3706	3766	61	-1			J
atp8	3767	3919	153	-	ATT	TAA	J
atp6	3907	4563	657	-13	ATG	TAA	J
cox3	4569	5346	778	5	ATG	T	J
trnG	5347	5409	63	-			J
nad3	5410	5763	354	-	ATA	TAA	J
trnA	5764	5825	62	-			J
trnR	5824	5888	65	-2			J
trnN	5889	5953	65	-			J
trnS1(ACN)	5953	6015	63	-1			J
trnE	6016	6077	62	-			J
trnF	6080	6148	69	2			N
nad5	6149	7811	1663	-	TTG	T	N
trnH	7812	7875	64	-			N
nad4	7879	9226	1348	3	ATT	T	N
nad4L	9207	9476	270	-20	ATT	TAA	N
trnT	9479	9541	63	2			J
trnP	9542	9604	63	-			N
nad6	9607	10090	484	2	ATA	T	J
cytb	10091	11227	1137	-	ATG	TAG	J
trnS2(UCN)	11226	11290	65	-2			J
nad1	11281	12201	921	-10	ATT	TAA	N
trnL1(CUN)	12202	12263	62	-			N
rrnL	12264	13443	1180	-			N
trnV	13444	13505	62	-			N
rrnS	13506	14245	740	-			N
CR	14246	16221	1,976	-			J

Table S4. Mtgenome organization of and *K. nativa.*

Name	Location		Size(bp)	Intergenic nucleotides	Codon		Strand
	From	To			Start	Stop	
trnI	1	66	66				J
trnQ	64	132	69	-3			N
trnM	135	205	71	2			J
nad2	206	1180	975	-	ATT	TAG	J
trnW	1179	1240	62	-2			J
trnC	1233	1295	63	-8			N
trnY	1296	1363	68	-			N
cox1	1362	2895	1534	-2	ATG	T	J
trnL2(UUR)	2896	2962	67	-			J
cox2	2963	3641	679	-	ATG	T	J
trnK	3642	3711	70	-			J
trnD	3711	3772	62	-1			J
atp8	3774	3926	153	1	ATC	TAA	J
atp6	3914	4570	657	-13	GTG	TAA	J
cox3	4574	5353	780	3	ATG	TAA	J
trnG	5352	5414	63	-2			J
nad3	5415	5765	351	-	ATT	TAA	J
trnA	5766	5826	61	-			J
trnR	5826	5888	63	-1			J
trnN	5890	5954	65	1			J
trnS1(ACN)	5954	6014	61	-1			J
trnE	6014	6076	63	-1			J
trnF	6077	6139	63	-			N
nad5	6140	7799	1660	-	ATT	T	N
trnH	7801	7863	63	1			N
nad4	7867	9214	1348	3	ATT	T	N
nad4L	9195	9464	270	-20	ATT	TAG	N
trnT	9467	9528	62	2			J
trnP	9529	9599	71	-			N
nad6	9602	10082	481	2	ATA	T	J
cytb	10083	11219	1137	-	ATG	TAG	J
trnS2(UCN)	11218	11285	68	-2			J
nad1	11276	12217	942	-10	ATT	TAA	N
trnL1(CUN)	12218	12280	63	-			N
rrnL	12281	13487	1207	-			N
trnV	13488	13548	61	-			N
rrnS	13549	14287	739	-			N
CR	14288	15716	1429	-			J

Table S5. Nucleotide composition and skewness comparison of different elements of four Hylicinae mtgenomes.

Species	Feature	Size(bp)	T%	C%	A%	G%	A+ T%	AT-skew	GC-skew
N. tuberculatus	PCGs	10950	42.8	12.0	33.2	12.0	76.0	-0.126	-0.003
	Control region	1479	37.8	11.0	41.0	10.1	78.8	0.041	-0.042
	tRNAs	1429	38.5	8.7	40.4	12.5	78.9	0.024	0.179
	rRNAs	1925	54.7	7.2	26.3	11.8	81.0	-0.351	0.240
	Whole genome	15737	38.1	15.2	47.1	9.5	77.1	0.222	-0.166
H. paradoxa	PCGs	10932	41.5	12.9	32.8	12.8	74.3	-0.118	-0.002
	Control region	531	40.3	7.2	46.3	6.2	76.6	0.070	-0.017
	tRNAs	1416	37.4	9.5	40.4	12.7	77.8	0.038	0.146
	rRNAs	1920	58.7	6.5	23.9	11.0	82.6	-0.307	0.312
	Whole genome	14762	28.1	15.2	47.7	9.1	75.8	0.259	-0.251
B. fujiana	PCGs	10947	43.2	11.3	34.0	11.5	77.2	-0.119	0.006
	Control region	1976	38.2	14.5	35.3	11.7	73.5	-0.039	0.104
	tRNAs	1416	37.6	9.2	40.5	12.8	78.0	0.037	0.164
	rRNAs	1920	58.7	6.5	23.8	11	82.5	-0.423	0.26
	Whole genome	16221	30.1	13.4	47.3	9.2	77.4	0.223	-0.185
K. nativa	PCGs	10962	40.3	13.5	32.7	13.5	73.0	-0.104	0.002
	Control region	1429	40.0	13.3	36.7	10.1	76.6	-0.043	-0.138
	tRNAs	1425	36.6	10.8	38.8	13.8	75.4	0.029	0.120
	rRNAs	1946	55.8	7.3	24.3	12.6	80.1	-0.394	0.268
	Whole genome	15716	27.1	15.8	47.3	9.8	74.4	0.272	-0.235

Table S6. Best partitioning schemes and models based on different datasets for Bayesian inference (BI) analysis.

Dataset	Partitioning scheme	Models
P123	P1: (*atp8*_pos2, *atp6*_pos1)	GTR+I+G
	P2: (*nad2*_pos2, *atp6*_pos2, *nad6*_pos2, *nad3*_pos2)	GTR+I+G
	P3: (*nad6*_pos3, *nad3*_pos3, *atp8*_pos3, *atp6*_pos3)	GTR+I+G
	P4: (*nad3*_pos1, *nad2*_pos1, *nad6*_pos1, *atp8*_pos1)	GTR+I+G
	P5: (*cox1*_pos1)	GTR+I+G
	P6: (*cox1*_pos2, *cox3*_pos2, *cytb*_pos2, *cox2*_pos2)	GTR+I+G
	P7: (*cox1*_pos3)	GTR+ G
	P8: (*cytb*_pos1, *cox3*_pos1, *cox2*_pos1)	GTR+I+G
	P9: (*cytb*_pos3, *cox2*_pos2, *cox3*_pos3)	GTR +G
	P10: (*nad1*_pos1, *nad4l*_pos1, *nad4*_pos1, *nad5*_pos1)	GTR+I+G
	P11: (*nad5*_pos2, *nad4*_pos2, *nad1*_pos2, *nad4L*_pos2)	GTR+I+G
	P12: (*nad1*_pos3)	GTR +G
	P13: (*nad2*_pos3)	GTR +G
	P14: (*nad5*_pos3, *nad4l*_pos3, *nad4*_pos3)	GTR +G
P123R	P1: (*atp6*_pos1)	GTR+I+G
	P2: (*cox2*_pos2, *atp6*_pos2, *cox3*_pos2,	GTR+I+G

	*nad3*_pos2, *cytb*_pos2)	
	P3: (*nad6*_pos3, *nad3*_pos3, *atp8*_pos3, *atp6*_pos3)	GTR+I+G
	P4: (*nad2*_pos1, *nad3*_pos1, *nad6*_pos1, *atp8*_pos1)	GTR+I+G
	P5: (*atp8*_pos2, *nad6*_pos2, *nad2*_pos2)	GTR+I+G
	P6: (*cox1*_pos1)	GTR+I+G
	P7: (*cox1*_pos2)	GTR+I+G
	P8: (*cox1*_pos3)	GTR+G
	P9: (*cytb*_pos1, *cox3*_pos1, *cox2*_pos1)	GTR+I+G
	P10: (*cytb*_pos3, *cox2*_pos3, *cox3*_pos3)	GTR+G
	P11: (*nad1*_pos1, *nad4l*_pos1, *nad4*_pos1, *nad5*_pos1)	GTR+I+G
	P12: (*nad5*_pos2, *nad4*_pos2, *nad1*_pos2, *nad4L*_pos2)	GTR+I+G
	P13: (*nad1*_pos3)	GTR+G
	P14: (*nad2*_pos3)	GTR+G
	P15: (*nad5*_pos3, *nad4l*_pos3, *nad4*_pos3)	GTR+G
	P16: (*rrnS*, *rrnL*)	GTR+I+G
AA	P1: (*atp8*, *nad3*, *nad6*, *cox3*, *nad2*, *cox2*, *cytb*, *atp6*)	MTREV+ I+G
	P2: (*cox1*)	MTART+ I+G
	P3: (*nad4l*, *nad4*, *nad5*, *nad1*)	MTART+ I+G

Table S7. Best partitioning schemes and models based on different datasets for maximum likelihood (ML) analysis.

Datase t	Partitioning scheme	Models
P123	P1: (*atp8*_pos2, *atp6*_pos1)	GTR+I+G
	P2: (*nad2*_pos2, *atp6*_pos2, *nad6*_pos2, *nad3*_pos2)	TVM+I+G
	P3: (*nad6*_pos3, *nad3*_pos3, *atp8*_pos3, *atp6*_pos3)	TRN+I+G
	P4: (*nad3*_pos1, *nad2*_pos1, *nad6*_pos1, *atp8*_pos1)	GTR+I+G
	P5: (*cox1*_pos1)	GTR+I+G
	P6: (*cox1*_pos2, *cox3*_pos2, *cytb*_pos2, *cox2*_pos2)	TVM+I+G
	P7: (*cox1*_pos3)	TIM+G
	P8: (*cytb*_pos1, *cox3*_pos1)	GTR+I+G
	P9: (*cytb*_pos3, *cox2*_pos3, *cox3*_pos3)	GTR+G

	P10: (*nad1*_pos1, *nad4l*_pos1, *nad4*_pos1, *nad5*_pos1)	GTR+I+G
	P11: (*nad5*_pos2, *nad4*_pos2, *nad1*_pos2, *nad4L*_pos2)	GTR+I+G
	P12: (*nad1*_pos3)	TIM+G
	P13: (*nad2*_pos3)	TRN+G
	P14: (*nad5*_pos3, *nad4l*_pos3, *nad4*_pos3)	K81UF+G
P123R	P1: (*atp6*_pos1)	GTR+I+G
	P2: (*cox2*_pos2, *atp6*_pos2, *cox3*_pos2, *nad3*_pos2)	TVM+I+G
	P3: (*nad6*_pos3, *nad3*_pos3, *atp8*_pos3, *atp6*_pos3)	TRN+I+G
	P4: (*nad2*_pos1, *nad3*_pos1, *nad6*_pos1, *atp8*_pos1)	GTR+I+G
	P5: (*atp8*_pos2, *nad6*_pos2, *nad2*_pos2)	TVM+I+G
	P6: (*cox1*_pos1)	GTR+I+G
	P7: (*cox1*_pos2)	TVM+I+G
	P8: (*cox1*_pos3)	TVM+G
	P9: (*cytb*_pos1, *cox3*_pos1, *cox2*_pos1)	GTR+I+G
	P10: (*cytb*_pos3, *cox2*_pos3, *cox3*_pos3)	GTR+G
	P11: (*nad1*_pos1, *nad4l*_pos1, *nad4*_pos1, *nad5*_pos1)	GTR+I+G
	P12: (*nad5*_pos2, *nad4*_pos2, *nad1*_pos2, *nad4L*_pos2)	GTR+I+G
	P13: (*nad1*_pos3)	K81UF+G
	P14: (*nad2*_pos3)	TRN+G
	P15: (*nad5*_pos3, *nad4l*_pos3, *nad4*_pos3)	K81UF+G
	P16: (*rrnS*, *rrnL*)	GTR+I+G
AA	P1: (*atp8*, *nad3*, *nad6*, *cox3*, *nad2*, *cox2*, *cytb*, *atp6*)	MTART+I+G +F
	P2: (*cox1*)	MTART+I+G
	P3: (*nad4l*, *nad4*, *nad5*, *nad1*)	MTART+I+G +F

Table S8. Detail of software and kit.

**Software/ Kit**	**Resourse**
Easy Pure Genomic DNA Kit	TransGen Biotech Co., Ltd, Beijing, China
NGS company	Illumina HiSeq Xten; Biomarker Technologies Corporation, Beijing, China
Geneious10.0.5	Biomatters, Auckland, New Zealand, http://www.geneious.com/
MITOS WebServer	http://mitos.bioinf.uni-leipzig.de/index.py
tRNAscan-SE Search Server v1.21	http://lowelab.ucsc.edu/tRNAscan-SE/
Tandem Repeats Finder server	http://tandem.bu.edu/trf/trf.html
CGView	http://stothard.afns.ualberta.ca/cgview_server
MEGA v 7.313	Penn State University, State College, PA, USA

Prism 6.01	GraphPad Software, San Diego, USA
MAFFT v 7.313 online service	https://mat.cbrc.jp/alignment/server/
GBlocks v 0.91b	http://molevol.cmima.csic.es/castresana/
	Gblocks/Gblocks_documentation.html
PartitionFinder 2.1.1	www.phylo.org
IQ-TREE v 1.6.8	http://www.iqtree.org/
MrBayes 3.2.6	www.phylo.org

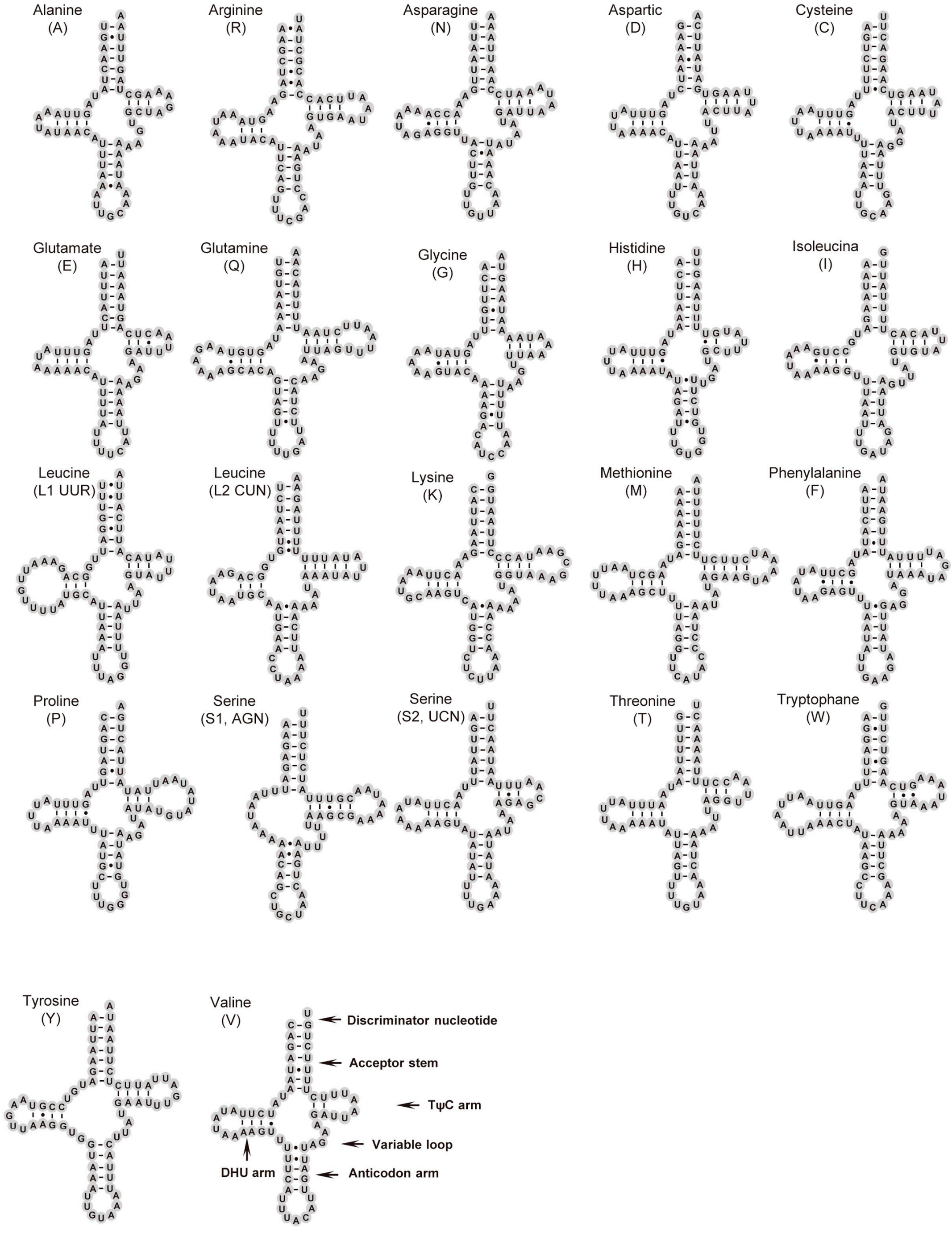

Figure S1. Predicted secondary cloverleaf structure for the tRNAs of *N. tuberculatus*.

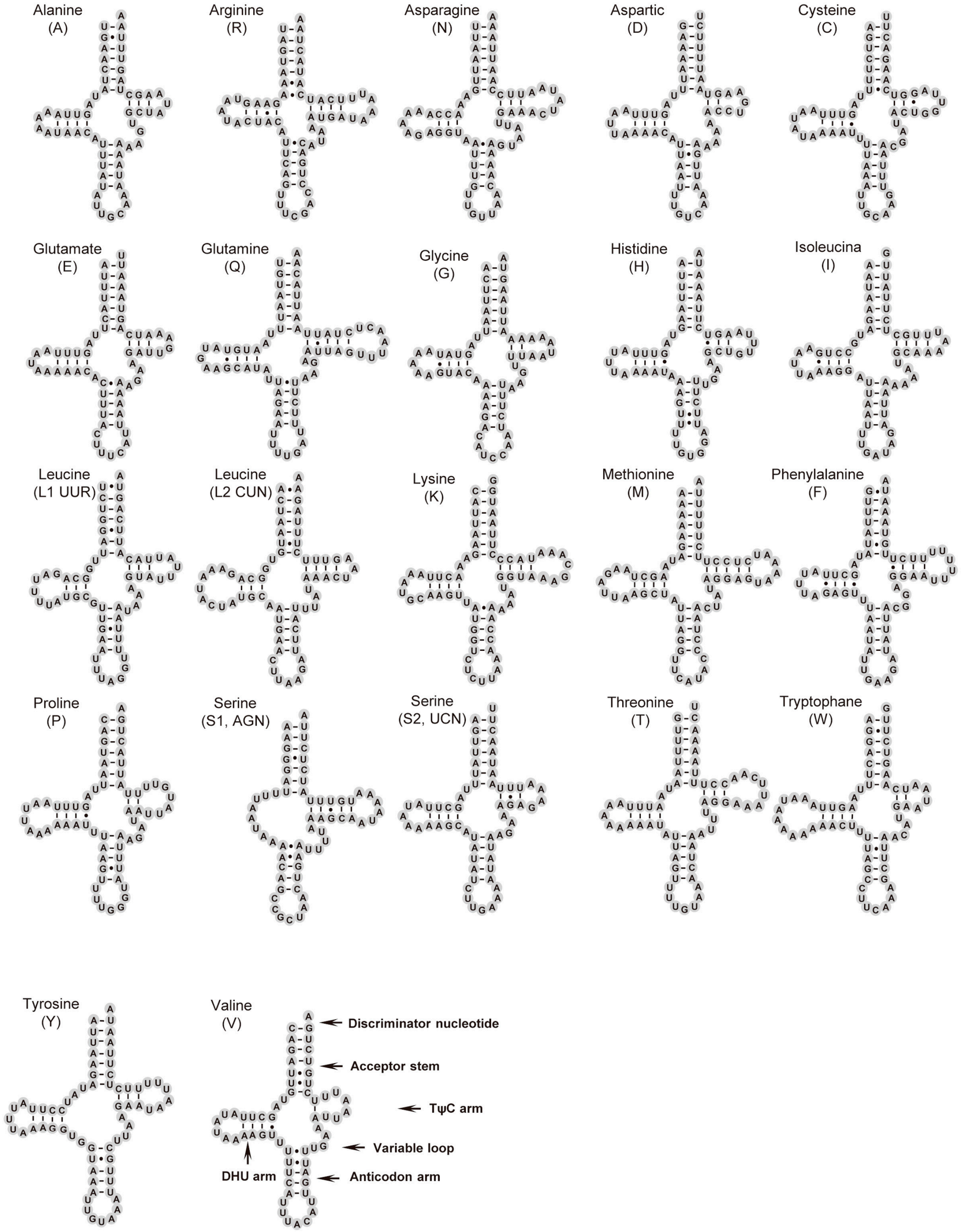

Figure S2. Predicted secondary cloverleaf structure for the tRNAs of *H. paradoxa.*

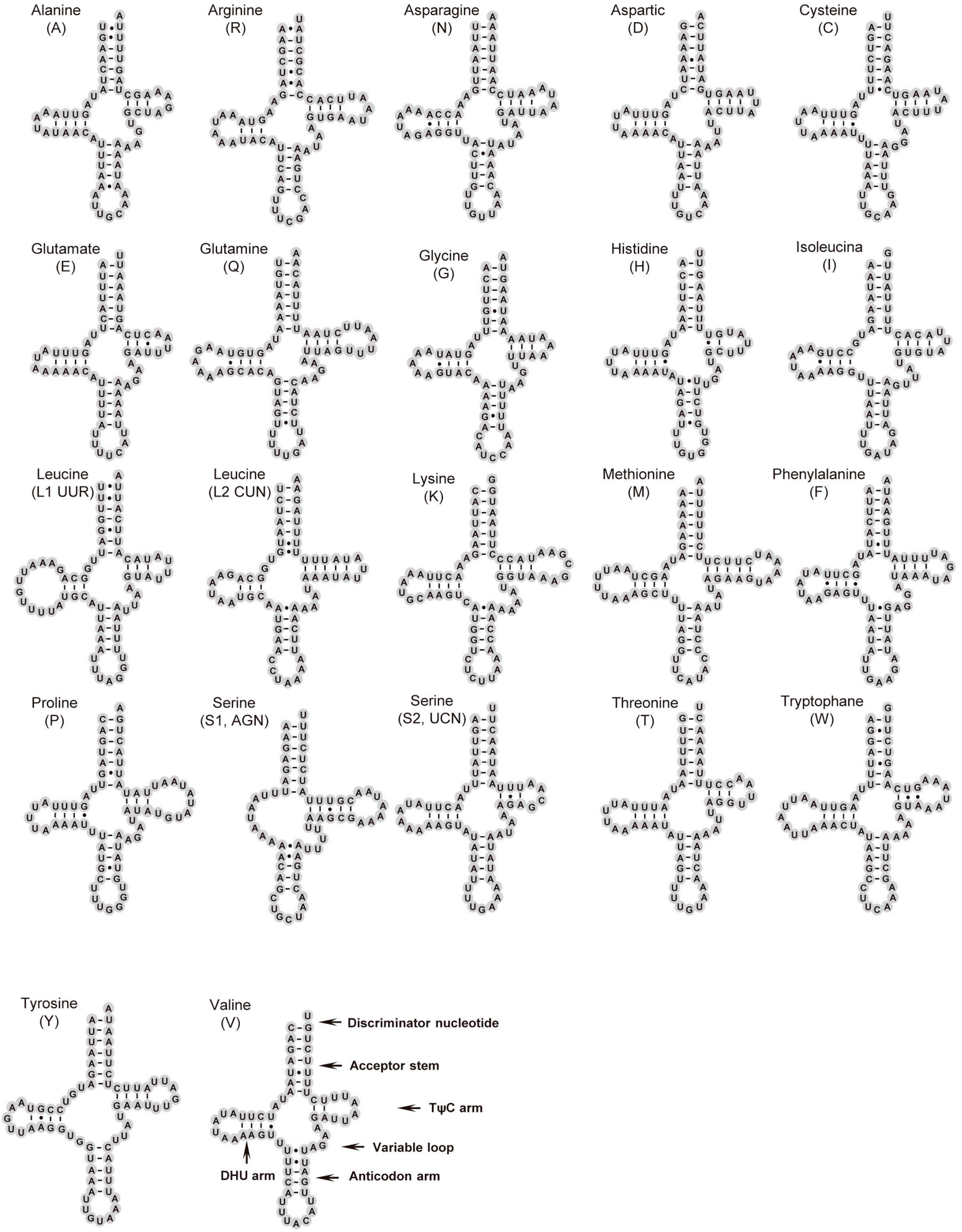

Figure S3. Predicted secondary cloverleaf structure for the tRNAs of *B. fujiana*.

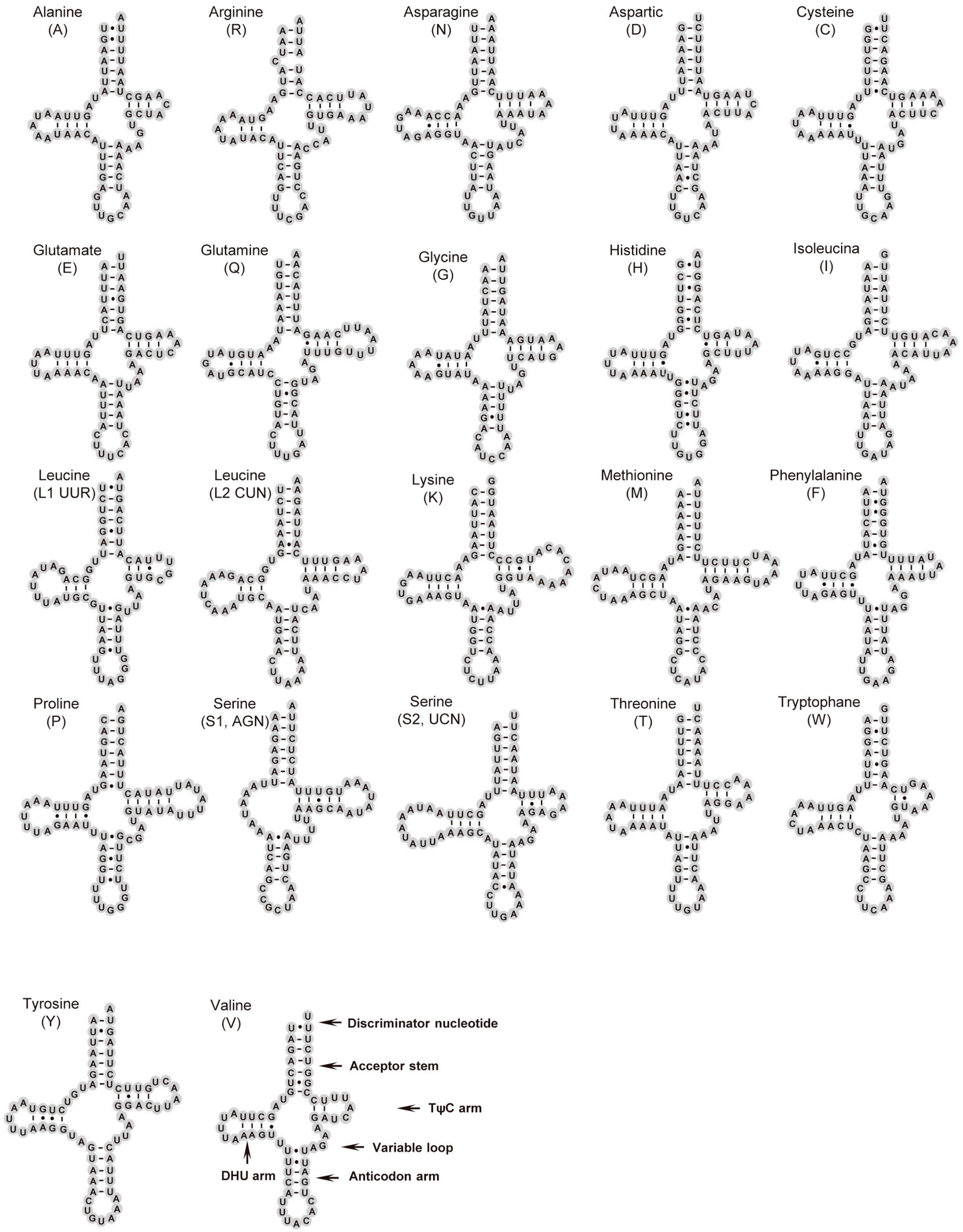

Figure S4. Predicted secondary cloverleaf structure for the tRNAs of *K. nativa*.
